# Sensing of Organic Vapors with Plasmonic Distributed Bragg Reflectors

**DOI:** 10.1021/acsami.5c02058

**Published:** 2025-04-24

**Authors:** Zdeněk Krtouš, Oleksandr Polonskyi, Pavel Pleskunov, Miroslav Cieslar, Bill Baloukas, Ludvik Martinu, Jaroslav Kousal

**Affiliations:** † Department of Macromolecular Physics, Faculty of Mathematics and Physics, Charles University, V Holešovičkách 2, 180 00 Prague, Czech Republic; ‡ Department of Engineering Physics, Polytechnique Montréal, Montreal, Quebec H3T 1J4, Canada; § Department of Chemical Engineering, University of California, Santa Barbara, California 93106-5080, United States; ∥ Department of Physics of Materials, Faculty of Mathematics and Physics, Charles University, Ke Karlovu 5, Prague 121 16, Czech Republic; ⊥ Department of Aerospace Engineering, Faculty of Mechanical Engineering, Czech Technical University in Prague, Karlovo náměstí 13, 121 35 Prague, Czech Republic

**Keywords:** nanocomposites, VOC sensing, distributed Bragg reflector, gas aggregation source, plasmonic nanoparticles

## Abstract

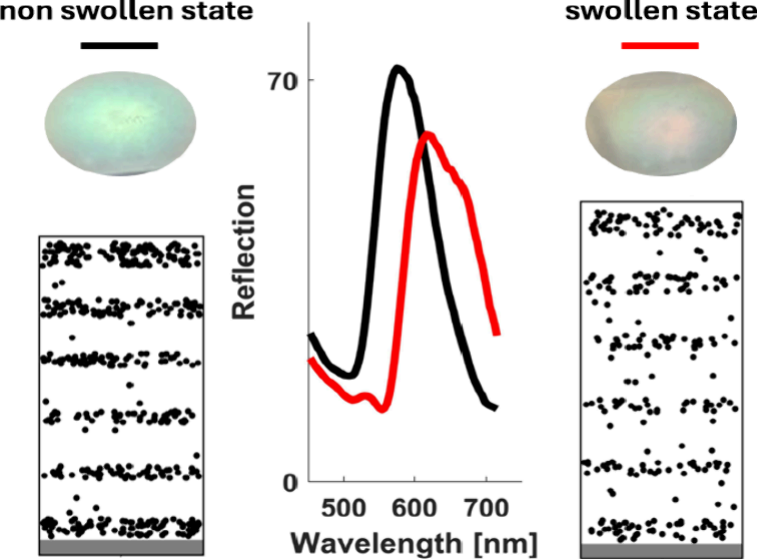

In recent years, advancements in air quality monitoring have been driven by the development of various sensor technologies, each with distinct advantages and limitations. Among these, polymer-based Distributed Bragg Reflectors (DBRs) have garnered significant interest for use in cost-effective, portable colorimetric sensors for detecting volatile organic compounds (VOCs). However, a key challenge in the fabrication of polymer-based DBRs lies in achieving an adequate refractive index contrast between the individual polymer layers. In this work, we fabricate plasmonic DBR sensors by a combination of low-temperature plasma-based techniques with reduced environmental footprint, investigate their potential as VOC sensors, and propose an optical model that links the sensors’ optical properties and microstructure. Plasmonic nanoparticles of silver (Ag) are synthesized by gas aggregation and embedded into thermally evaporated poly(lactic acid) (PLA) layers to create nanocomposites with an enhanced refractive index (∼2.0). A 6-bilayer plasmonic DBR sensor is then produced by alternating depositions of plain PLA and nanocomposite layers as low and high refractive index materials, respectively. The resulting DBR achieves a 77% reflectance at 570 nm. The potential use-case of such a DBR as a VOC sensor is highlighted by its optical response upon exposure to ethanol (a model VOC) vapors as well as other VOCs (water, propanol, acetone, hexane). In an ethanol atmosphere, swelling of the polymer layers occurs, resulting in a red-shift of the reflection peak to 640 nm and a change in the DBR color. We take advantage of a generalized Maxwell-Garnett approach to create an advanced model that accurately reproduces the DBR spectra and captures swelling and degradation by accounting for structural changes and the behavior of isolated and coalesced Ag NPs within individual layers. Despite a decrease in the sensing performance with the number of swelling cycles, these plasmonic DBRs offer a promising solution for low-cost real-time VOC sensing.

## 1 Introduction

Alcohols such as ethanol, *n*-propanol, isopropyl alcohol, and *n*-butanol are commonly used volatile organic compounds (VOCs). Ethanol, widely used in the chemical industry, can lead to adverse health effects, including nausea, headache, dizziness, and even cancer in cases of prolonged exposure. VOCs are characterized by high vapor pressures at room temperature and limited dispersibility, increasing the likelihood of prolonged exposure and associated health risks. Recently, 1-D photonic crystals have been investigated for VOC sensing applications. One of the type of such sensors is based on polymer-based distributed Bragg reflectors (DBRs).^1−5^ These DBRs operate by swelling upon exposure to VOC vapors, increasing layer thickness, altering interference, and changing the reflector’s color. The proposed sensing mechanism relies on polymer swelling in the presence of VOCs and can be described by the Flory–Huggins theory.^6,7^

A key challenge in fabricating polymer-based DBRs is the limited refractive index contrast commonly found for compatible polymers. Refractive indices of polymers range from about 1.3^8,9^ to around 2.0,^9^ but coprocessing these materials is often constrained by their different chemical properties. To enhance the refractive index contrast, doping polymers with high-refractive-index nanoparticles has been proposed.^10−12^ For example, Lova et al.^13,14^ have reported using DBRs for toluene sensing, combining cellulose acetate (*n* ≈ 1.46) and polystyrene (*n* ≈ 1.57) doped with high-index ZnO nanoparticles (*n* ≈ 1.98). The 15-bilayer DBR achieved 45% reflection at 1500 nm. However, the refractive index of the composite with a 2.5% ZnO volume increased the index only slightly, reaching *n* = 1.587 at 800 nm compared to *n* = 1.581 at the same wavelength for polystyrene.

Although a significant increase of refractive index with dielectric nanoparticles (typically, metal oxides such as ZnO, TiO_2_, etc.) is possible,^10,11^ it typically requires very high filling factors. An alternative approach is envisioned in embedding metallic nanoparticles (Au, Ag, Cu)^9,15,16^ instead of their dielectric counterparts. Convertino et al.^17−19^ developed acetone-responsive DBRs using alternating layers of bare tetrafluoroethylene (*n* ≈ 1.3) and gold-doped tetrafluoroethylene composites (*n* ≈ 1.7) deposited by cosputtering the gold and polymer. This four-bilayer structure achieved 75% reflection at 1600 nm owing to the higher refractive index contrast attained through the plasmonic properties of gold. Indeed, gold nanoparticles exhibit localized surface plasmon resonance (LSPR), the coherent oscillations of conductive electrons driven by a propagating electromagnetic wave, creating a sharp absorption peak around 530 nm in a matrix with a refractive index of 1.3. From the Kramers–Kronig relations, it can be inferred that an LSPR induces an upsurge in the refractive index which is red-shifted in terms of wavelength with respect to the extinction coefficient’s peak position. The amplitude of the index change allows one to reach much higher effective refractive indices for the NP-containing layers. A common disadvantage of this approach, however, is the significant absorption and nonlinear response of the plasmonic nanocomposites.

Nonetheless, this limitation can be bypassed by carefully designing the composite coatings while considering the absorption’s contribution. For example, Convertino et al.^17−19^ designed a DBR with a central wavelength far from the LSPR peak at 1600 nm, where absorption was relatively low and, thus, change of refractive index in the IR region was minor. Sun et al.^20^ demonstrated that Au nanoparticle-based DBRs, fabricated by implanting Au in a SiO_2_ matrix, could achieve reflection peaks near the LSPR peak (540 nm for a SiO_2_ matrix with a refractive index of 1.45) at 550 nm. However, silver (Ag) nanocomposites, with an LSPR peak at 400 nm, may be preferable for visible-region DBRs, as a larger part of the visible spectra can be rendered with minimized absorption and reflection peak at lower wavelengths can be targeted compared to gold-based nanocomposites. For example, Schürmann et al.^15,21^ demonstrated a PTFE-silver composite DBR deposited via cosputtering of polymer and silver, achieving an 85% reflection peak. This reflector was designed with its reflection peak in the near IR at 850 nm.

In all of the examples discussed above, the effective refractive index of the composite layers was typically estimated as an approximate value. For VOC-sensing DBR synthesis, precise refractive index values are not essential since functionality, rather than optimal optical performance, is the priority. However, accurate optical modeling can provide valuable insights into the sensing mechanism. In the case where scattering can be neglected, nanocomposites are generally modeled using effective medium theories such as Maxwell-Garnett (MG-EMA) and Bruggeman (B-EMA). However, real composites often deviate from these simple models due to nanoparticle size effects and near-field interactions between the nanoparticles, which lead to LSPR peak broadening and shifting. Therefore, modified versions of MG-EMA and B-EMA, which include appropriate corrections,^22−26^ are being developed. Vieaud et al.^27^ demonstrated that Au-polymer composites could be accurately modeled via a generalized Maxwell-Garnett (GMG-EMA) model, capable of describing plasmonic composites with relatively large nanoparticles (20 nm diameter) and dimers thereof.

In the present work, we fabricate a DBR by embedding Ag NPs into composites with a poly(lactic acid) (PLA)-like matrix. Unlike previous approaches, we incorporate the NPs synthesized using a gas aggregation cluster source (GAS). This approach leads to a similar structure of Ag inclusions embedded in the polymer matrix; however, in comparison to conventional magnetron sputtering, the structural properties are different. In fact, GAS typically produces larger and more spherical nanoparticles than sputtered or implanted inclusions; therefore, the optical properties (LSPR shift as well as broadness) are different for the GAS composites.^28^ We demonstrate that the composites fabricated using GAS are a better match to the GMG-EMA model, which opens a pathway for precise optical investigation of the DBRs. We use the GMG-EMA model for the in operando optical characterization of the DBR during sensing tests and for ex situ analysis to examine its state before and after the tests.

## 2 Materials and Methods

### 2.1 DBR Synthesis

The DBR consisted of a stack of PLA-like plasma polymer layers and nanocomposite layers, the latter being based on a matrix of the same base PLA material with the addition of Ag nanoparticles. The synthesis process of the reflector is based on a combination of two vacuum-based thin-film deposition techniques. The plasma polymer layers, which form the low refractive index layers of the DBR, were deposited using Plasma-Assisted Vapor Thermal Deposition with a continuous feed (PAVTD) setup,^29^ using a polylactic acid (PLA) 3D printing filament (natural PLA, Gembird) fed at a rate of 2 g/h used as a precursor polymer. The polymer is heated in a copper crucible (volume of 5 mL) to 300 °C, which leads to its stable evaporation. A constant deposition rate in the range of several hours is achieved by continuously refilling the crucible. The deposition chamber was maintained at an argon pressure of 0.5 Pa, while a plasma discharge, operating at a 13.56 MHz RF power of 5 W, was used to induce “mild” plasma polymerization within the film described here as PLA-like. Our previous works^30,31^ show that such conditions result in formation of a polymer characterized by a limited degree of cross-linking.

**Figure 1:**
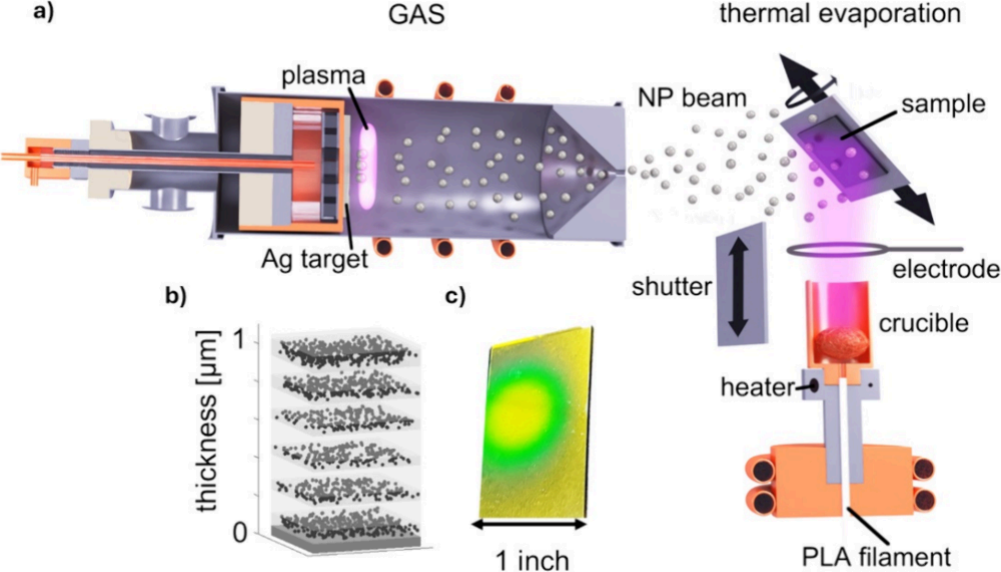
(a) Schematics of the implemented deposition setup, (b) schematic representation of the structure of the deposited reflectors, and (c) photo of one such deposited reflector.

The metal–polymer nanocomposite layers, which form the high refractive index layers of the DBR, were formed by codepositing PLA-like polymer material, as described above, with Ag NPs generated by a GAS. The GAS system utilized a DC magnetron with a 3 in. Ag target, operating at a current of 500 mA and an Ar pressure of 70 Pa. The working principle of the GAS is described in detail in our previous publications.^32,33^ For a schematic representation of the deposition setup, see Figure 1a.

The reflectors were then prepared by depositing alternating layers of the polymer and the polymer composite containing silver nanoparticles. The structure, schematically shown in Figure 1b, of the individual layers was controlled by maintaining a constant deposition rate of the polymer and switching the cluster source on and off at periodic intervals, giving rise to a multilayer stack of alternating high and low refractive index layers, each with approximately quarter-wave (QW) thicknesses.
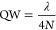
1

By stacking multiple QWs one on top of the other, a higher reflectivity is achieved through constructive interference resulting in a drop of light transmitted through the structure.^34^ Note that, in the case of traditional dielectric reflectors, the term “stopband” refers to the wavelength region where light is not transmitted through the reflector. However, in the present case, this term is not applicable as the plasmonic silver nanoparticles exhibit a dominant absorption in both the short-wavelength region (around 400 nm) and the long-wavelength region (around 700 nm). As a result, our reflectors are largely opaque across the entire visible spectrum. A photo of one such reflector is shown in Figure 1c.

This modifies the QW interference conditions, as the standard DBR equations do not fully apply to absorptive media. Therefore, the DBR design was verified and optimized by using the Transfer Matrix Method (TMM). The effective refractive indices of the composite and polymer layers were estimated by using a further defined optical model. The deposition process was optimized to achieve polymer layer thicknesses (*t*_pl_) around 100 nm and composite layer thicknesses (*t*_cl_) of approximately 70 nm. The refractive index of the polymer layer, namely, the PLA material, is approximately *n*_550_ ≈ 1.46. The refractive index of the composite layer can be tuned by modifying the number of embedded NPs. Due to the dispersive nature of the composite layers and the limited precision in controlling the number of embedded NPs, we estimate the effective refractive index using the spectral range between 500 and 600 nm. This range is characterized by a relatively low and stable dispersion and is close to the primary wavelength of the DBR. The effective refractive index is determined to be within the range 1.95 ≤ *n* ≤ 2.10.

### 2.2 DBR Characterization and Swelling Test

The morphology and structure of the NPs embedded in a composite layer deposited onto a Si_3_N_4_ membrane were studied by transmission electron microscopy (TEM, 2200FS, Jeol Ltd.) with a FEG cathode operated at 200 kV. The DBR structure was characterized using scanning electron microscopy (SEM, Jeol JSM-7900F) by imaging its cross-section when deposited on a Si substrate. The size distribution of the nanoparticles was determined using an in-house developed Solarius software for nanoparticle counting on the TEM as well as cross-sectional SEM images. The DBR’s reflectivity and transmission (nonpolarized light) were measured on a glass substrate using a Cary 7000 spectrophotometer equipped with a universal measurement accessory (UMA) at an incidence angle of 6 degrees, over the spectral range of 250 to 2500 nm.

To investigate the sensing properties of the DBR, the reflector was placed into a 3D-printed gas flow cell with an internal volume of 350 mL for reflectance measurements at an incidence angle of 9 degrees. This flow cell was used to monitor changes in the DBR’s reflectance spectra in response to atmospheric gas admixtures. Ethanol vapor was selected as the model gas for this study. The concentration of ethanol in the carrier gas (Ar) was controlled by splitting the gas flow into two lines: one carrying pure Ar at a flow rate of 0.25 L STP/min and the other passing through a bubbler containing ethanol at a flow rate of 0.1 L STP/min, resulting in an ethanol vapor concentration of approximately 2%. The vapor pressure of ethanol at room temperature is 8.7 kPa.^35^

The ethanol flow was regulated by a valve positioned before the bubbler, with a timed cycle of 3 min on and 3 min off. Throughout the entire sensing test, reflectance measurements were taken every second using an Ossila Optical spectrophotometer, covering the range from 400 to 750 nm. A silver mirror was used as a reference to calibrate the intensity of the LED lamp with the spectrophotometer. The schematic of the sensing setup is shown in Figure S1.

Finally, the reflectivity and transmission were once again measured after ethanol sensing using the Cary 7000 spectrophotometer, covering the full spectral range of 250 to 2500 nm, both in transmission and reflection.

### 2.3 Optical Model

The spectral simulations were conducted using a custom MATLAB script based on the TMM. Our code follows the implementation of the TMM in the *OpenFilters* software.^36^ The optical properties of the composites were modeled using the Generalized Maxwell-Garnett (GMG) theory, the implementation of which follows.^37^ The effective dielectric function of a composite is defined as
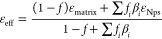
2where *f*_*i*_ is the volumetric filling factor of inclusions with shape factors β_*i*_ and *f* is the total volumetric filling factor of all inclusions. The dielectric function ε_NPS_ is a dielectric function of a nanoparticle material, while the dielectric function ε_matrix_ is a dielectric function of the host environment. This approach differs from the classical Maxwell-Garnett (MG) theory by summing over various types of inclusions, each weighted by a depolarization factor (β), also known as the shape factor. While MG theory describes the optical response of plasmonic nanocomposites with a low inclusion filling factor—assuming isolated spheres embedded in a dielectric medium with no interaction between particles—GMG extends this to account for short-range interactions, approximating these effects by ellipsoids.^27,37^ This generalization allows for a more accurate description of nanocomposites with higher filling factors, which is necessary for the investigated Bragg reflectors.

In our modeling of distributed Bragg reflectors (DBRs), we use a GMG with two components. The first component represents isolated spherical NPs, where the β factor corresponds to that of the classical MG theory for spherical NPs.
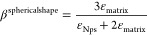
3

The second component represents ellipsoidal NPs, where the β factor accounts for the interaction between the touching particles in the composites.
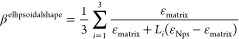
4

For ellipsoids stretched in one dimension (i.e., prolate spheroids), the relationship among the geometric factors *L*_1_, *L*_2_, and *L*_3_ follows the following dependence:
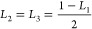
5

A similar approach for fitting plasmonic composites with the GMG-EMA and approximating dimer nanoparticles with ellipsoids has been used in^27^ for gold nanoparticles embedded in polymer films. In comparison, with other publications,^27,37^ we limit the model to prolate particles as a means of representing dimers and do not include oblate and general ellipsoids. On the other hand, we do allow for ellipsoids with varying degrees of elongation to capture particles with different levels of coalescence. The fitted distribution *P*(*L*_1_) follows a normal distribution:
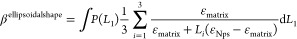
6

The optical properties of silver were modeled using the Lorentz–Drude model,
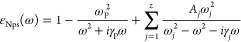
7where ω_P_ is the plasma frequency associated with intraband transitions, with a damping constant γ_P_, and *z* is the number of oscillators necessary to describe the interband part of the dielectric constant, each with a resonant frequency ω_*j*_, bandwidth γ_*j*_, and strength *A*_*j*_. The parameters of Drude–Lorentz oscillators were taken from ref (38).

The refractive index of the polymer matrix was measured by using spectroscopic ellipsometry and fitted by using a Cauchy model. The optical properties of the polymer were used as a matrix property in the case of the nanocomposite. The PLA has a refractive index in the range of 1.47–1.45 in the whole considered spectral range (350–2500 nm) while having negligible absorption.

## 3 Results and Discussion

### 3.1 Nanocomposites and DBR Design

**Figure 2:**
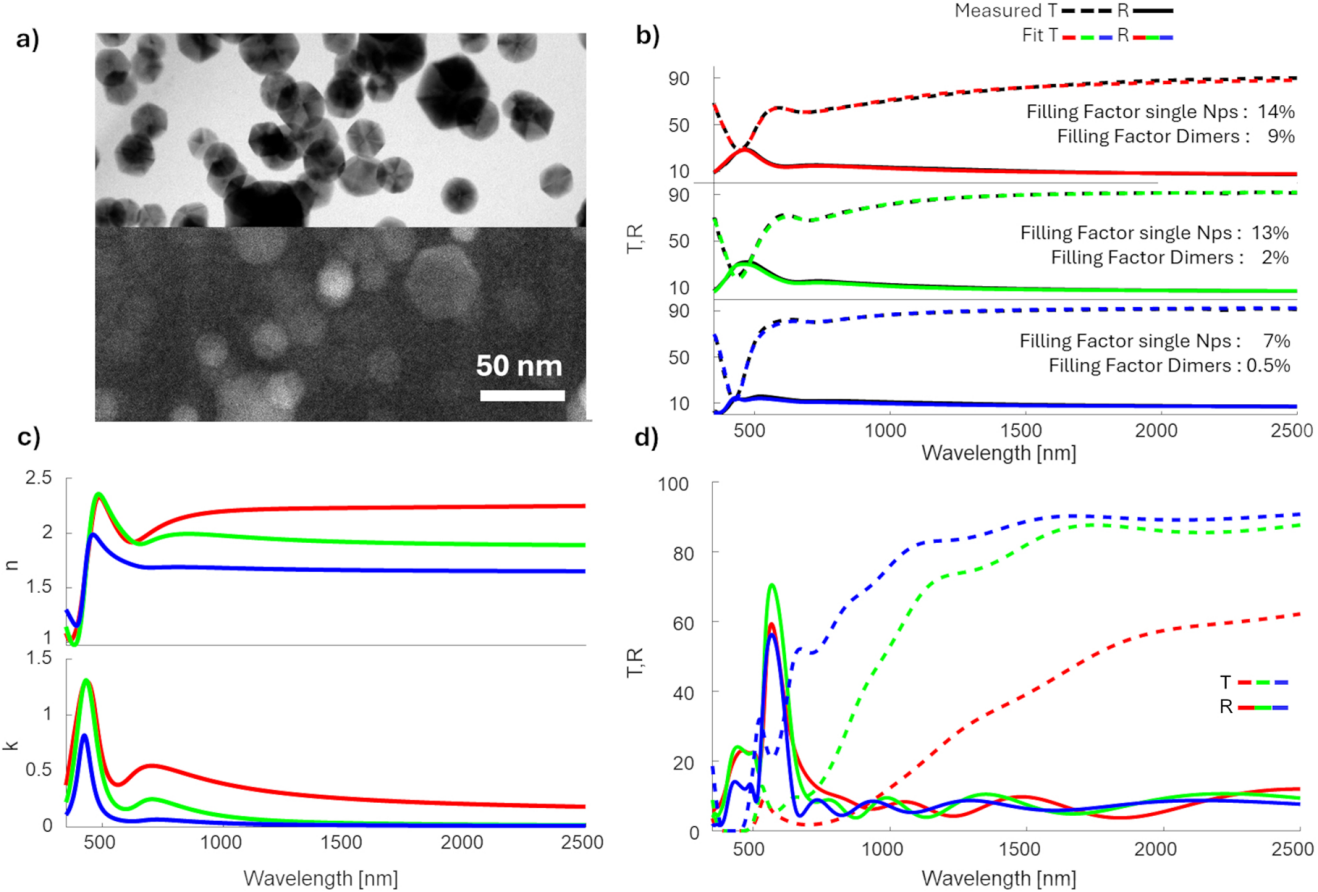
(a) TEM image of the composite layer, top: Secondary Electron Imaging mode, bottom: Bright Field mode, (b) T and R spectra (black lines) fitted with the GMG-EMA optical model (colored lines) for three different filling factors, (c) *n* (top), *k* (bottom) of the fitted composites, (d) designed DBR (central wavelength 570) for *n* and *k* of the investigated composites.

The first step in the rational design of multilayered optical filters involves understanding the optical and structural properties of the individual layers. We begin by analyzing the size and morphology of Ag NPs in the composite layer using TEM. The analysis was performed on an 80 nm-thick composite containing Ag NPs with an approximate volumetric filling factor (FF) of 15%. Figure 2a shows the Bright Field (BF) micrograph captured on the nanocomposite layers in a top-view orientation. We estimate the average NP diameter to be 24 ± 7 nm. Furthermore, the image indicates the presence of NP dimers and chains within the composite. However, since the TEM image represents a 2D projection of a 3D structure, a precise quantitative assessment of coalesced particles is challenging. This limitation is addressed by imaging the same region using an in-lens secondary electron detector (SEI) providing topological contrast (bottom part of Figure 2a). The SEI confirms the presence of dimers and aggregated NPs.

The localized surface plasmon resonance (LSPR) properties of agglomerated particles differ from those of isolated nanoparticles due to the excitation of dipole and higher-order plasmonic modes.^39−41^ This effect can be modeled using the Effective Medium Theory, approximating dimers as ellipsoidal particles, as discussed in the previous section. To evaluate the validity of this model, a series of nanocomposites with varying filling factors were prepared. The transmission (*T*) and reflection (*R*) spectra of the composites deposited on glass substrates are shown in Figure 2b. The colored lines in Figure 2b represent optical fits to these spectra using the GMG-EMA model.

The optical model accurately reproduces the measured spectra by describing the LSPR of individual Ag NPs at 400 nm and a secondary absorption peak originating from dimers and chains at around 700 nm. Additionally, the model provides quantitative insights into the distribution of isolated and coalesced NPs. For the low FF composite (FF = 8%), most NPs remain separated; the chaining and agglomeration become more pronounced for higher FF composites (15 and 23%). Here, a larger fraction of NPs contribute to dimers, chains, or agglomerates, respectively. Figure 2c shows the refractive index (*n*) and extinction coefficient (*k*) of the composite layers as modeled by GMG-EMA. The refractive index increases significantly compared to the plain polymer matrix (*n* ≈ 1.46) for both low (8%) and medium (15%) filling factors. However, further increasing the filling factor to 23% does not lead to a significant increase in the refractive index in the visible spectrum.

For the design of DBRs, achieving a high refractive index contrast while minimizing absorption is crucial. Figure 2d presents a DBR design based on the layers discussed earlier. This design employs a 6-bilayer structure optimized for maximum reflectivity at a central wavelength of 570 nm. The green-yellow spectral region (500–600 nm) was selected for optimization because LSPR absorption constrains the blue region (350–500 nm), while the red region (600–750 nm) is allocated for VOC sensing due to reflector swelling. This approach ensures that the reflector operates in the visible range, facilitating straightforward interpretation of its response to ethanol as a sensor. Under these design conditions, the optimal filling factor of the composite layers is approximately 15%. At a lower filling factor (8%), the number of layers is insufficient to fully reflect light at the central wavelength, leading to significant light transmission through the DBR. Conversely, at a higher filling factor (23%), increased absorption in the composite layers reduces the DBR’s reflectivity. Thus, a balance between refractive index contrast and absorption is achieved at 15% filling factor, making it the most suitable for effective reflector performance.

### 3.2 DBR Synthesis

**Figure 3:**
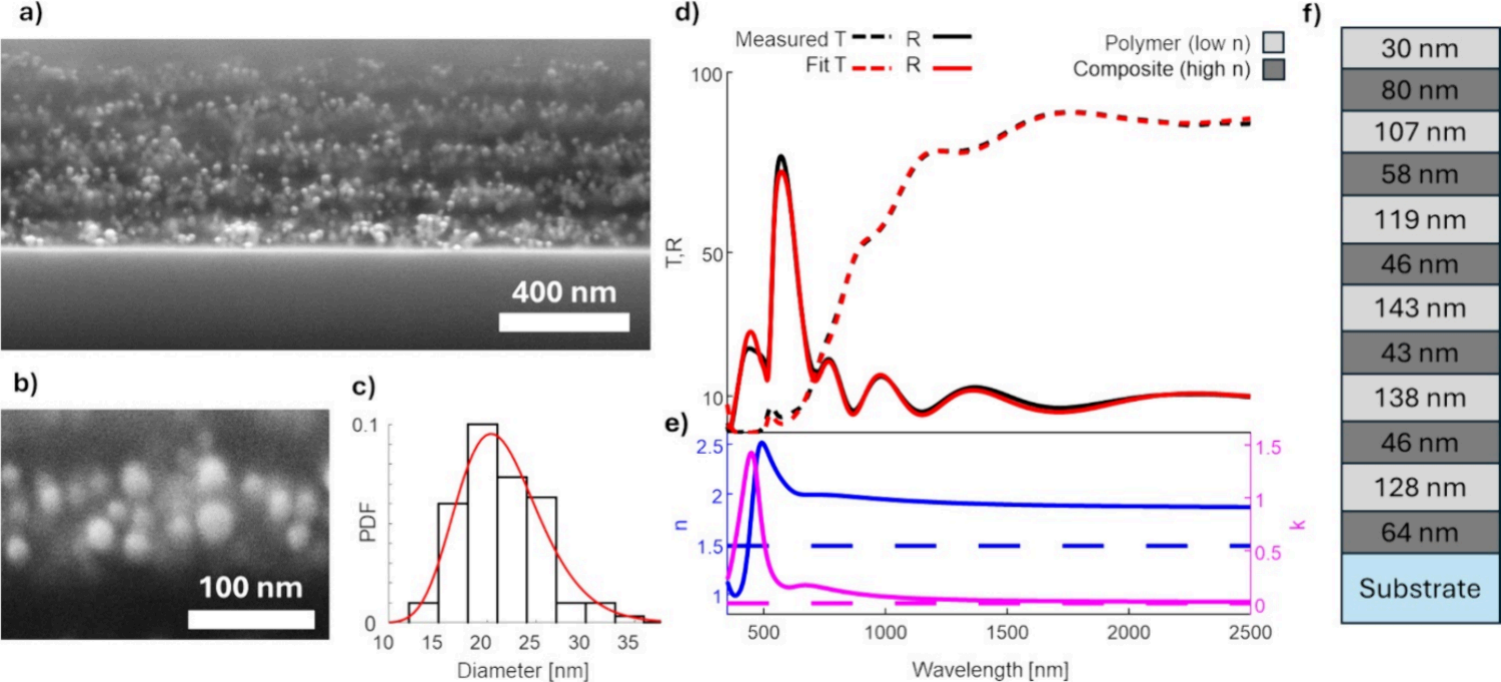
(a) SEM image of the cross-section of a DBR with 10 layers; (b) Micrograph of a larger magnification showing the microstructure of the composite layer—Ag NPs in PLA; (c) size distribution of the nanoparticles. (d) Transmittance (*T*) and reflectance (*R*) of a DBR with 12 layers; black lines: measured *T* and *R*; red lines: *T* and *R* fitted with the GMG-EMA optical model; (e) blue lines: refractive index (*n*) of the composite (solid line) and polymer (dashed line); magenta lines: extinction coefficient (*k*) of the composite (solid line) and polymer (dashed line), derived from the optical model. (f) Fitted structure of the reflector.

The DBR was synthesized with respect to the optimization described in the previous section. The structure of the reflector is shown in Figure 3a. The SEM image reveals a granular structure of the composite, with additional details highlighted in the close-up view in Figure 3b. The NP size distribution, shown in Figure 3c, follows a log-normal distribution with a mean diameter of 22 ± 5 nm, consistent with the size distributions achieved for the composite films discussed in the previous section. Since the average nanoparticle diameter is approximately one-third of the nanocomposite layer thickness, randomly protruding nanoparticles create a transitional interlayer between the polymer and nanocomposite layers. As a result, the multilayered structure in the SEM image appears to lack abrupt interfaces, exhibiting a structurally blurred appearance. Nevertheless, the composite and polymer layers remain distinguishable.

In practice, the reflector structure resembles a rugate filter, essentially a DBR with a continuous periodically varying refractive index gradient. For modeling purposes, however, it is more practical to represent the structure with well-defined layers as this approach simplifies implementation and interpretation by reducing the number of fitted parameters. The optical model, discussed in detail later, indicates that this structural blurring has a minimal impact on the optical performance.

The *R* and *T* spectra (black lines) of the reflector, consisting of 6 composite layers and 6 polymer layers deposited on a glass substrate, are shown in Figure 3d. The maximum reflectance peak occurs at 570 nm with a reflection of 77%. The fitted transmission and reflection spectra are shown in Figure 3d as red lines. The refractive index *n* and extinction coefficient *k* of the composite layers are shown in Figure 3e. The *n* and *k* of the DBR composite layers match the composite layer with a filling factor of 15%, as described in the previous section. The extinction coefficient exhibits a narrow LPSR peak characteristic of silver nanoparticles at 400 nm as well as the presence of a secondary peak originating from dimers in the 600 to 1000 nm region; the presence of this tail in *k* needs to be taken into account to correctly predict the performance of the DBR during the fitting process as it absorbs in this spectral range, similarly as the previously discussed composite films. Such an effect could not be modeled using the standard Maxwell-Garnet model, as shown in Figure S2.

In the optical model, we assume that all layers have the same filling factor of Ag NPs and that there is a well-defined interface between the composite and the polymer layers. Although neither of these assumptions is fully accurate, this simplified model still provides a good description of the optical response of the reflector. The fitted thicknesses of the individual reflector layers are presented in Figure 3f. The total filling factor corresponds to the 16% filling factor of silver in composite layers.

### 3.3 DBR as a Sensor—Ethanol Cycling

Since the primary application of this DBR is in volatile organic compound (VOC) sensing, we were particularly interested in examining its optical response when exposed to a model volatile gas. The swelling of the DBR increases the thickness of the structure, altering interference conditions and shifting the central reflection wavelength toward the red.^17^ For demonstration purposes, ethanol was chosen as the model VOC for this study, because the PLA-like layers in the DBR are partially ethanol-soluble, suggesting a potentially significant response.

**Figure 4:**
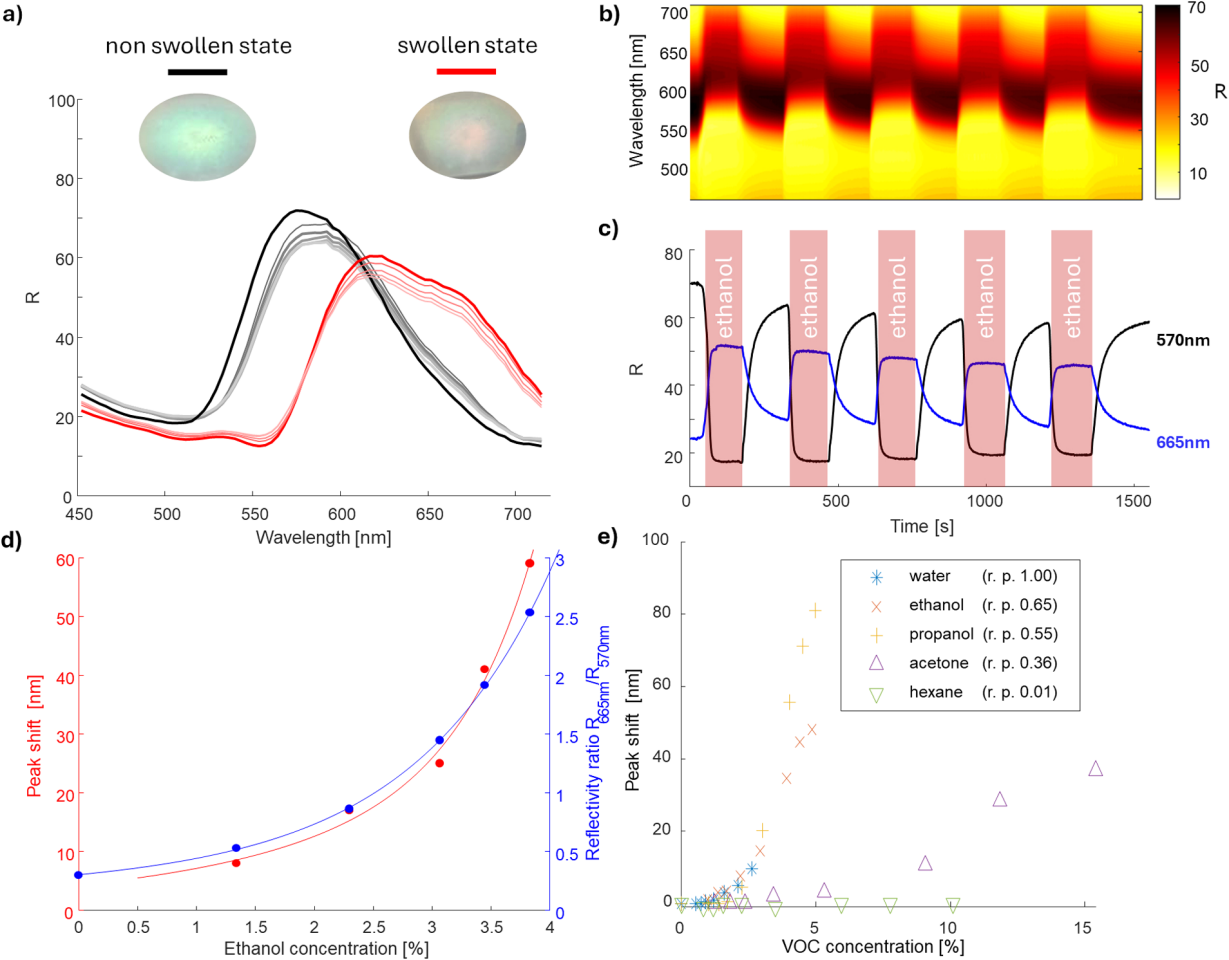
(a) Reflection spectra of the DBR in its original (black) and swollen (red) states. Insets: photographs (spot size approximately 1 in.) of the reflectors in the nonswollen and swollen states. (b) In-situ reflection spectra obtained during cycling in the 450 to 750 nm range and (c) in situ reflection during cycling at key wavelengths of interest, 570 and 665 nm. (d) Peak reflectance shift of the DBR and 665 nm/570 nm reflectance ratio as a function of ethanol concentration. (e) Dependence of the peak reflectance on the DBR shift for different Volatile organic compounds (water, ethanol, propanol, acetone, hexane).

The *R* spectrum measured before ethanol exposure is shown as a thick black line in Figure 4a. Ethanol vapor at a 2% concentration in an Ar carrier gas was introduced in cycles of 3 min of exposure followed by 3 min of recovery. The spectrum after full swelling during the first exposure cycle is presented as a thick red line in Figure 4a, while spectra for each subsequent relaxed and swollen state are shown as thin lines of corresponding colors. Photographs of the reflector, taken after 20 cycles, are included as the insets in Figure 4a.

The spectra and sample photos indicate a strong response of DBR to ethanol vapors. The reflection peak shifts from 570 nm in the nonswollen state (green) to 640 nm (red) in the swollen state. Figure 4b shows the time-dependent reflection spectra, while Figure 4c displays the optical response at selected wavelengths of 570 and 665 nm. These spectral lines were chosen for optimal sensitivity. Although they do not coincide with the reflection maxima in the swollen and nonswollen states, the relative difference between the maximum and minimum reflectance in these states is greatest at these wavelengths.

The reflector’s response to ethanol exposure is nearly instantaneous, reaching full swelling in approximately 20 s, while the deswelling process is significantly slower. In fact, the purge time of 3 min was not sufficient to allow the reflector to come back to a fully nonswollen state. This can explain, in part, why the DBR fails to regain its initial reflectance after the first cycle. The sensing performance of the DBR is also dependent on the ethanol concentration in the mixture. As the ethanol concentration increases, the reflection peak shifts progressively toward to higher wavelengths, as shown in Figure 4d. Both the peak wavelength shift and the intensity ratio between two selected wavelengths (570 and 665 nm) follow an exponential trend with respect to ethanol concentration.

To assess the chemical selectivity of the DBR sensor, we exposed it to vapors of solvents with varying relative polarities ranging from 0.01 (nonpolar, model substance—hexane) to 1.00 (highly polar, model substance—water), as shown in Figure 4e. The strongest responses were observed with ethanol (0.65) and propanol (0.55), where the reflectance peak shifted by 20 nm at a 3% methanol concentration and 15 nm at a 3% ethanol concentration. Water vapor (1.00) induced a moderate response (12 nm at 3%), while acetone (0.36) produced only a weak effect (2 nm at 3%). In contrast, exposure to hexane (0.01) resulted in no detectable sensor response. Initial signs of saturation were observed after a reflectance shift of approximately 40–60 nm for the ethanol vapors. Interestingly, this saturation was absent during the first ethanol exposure tests, suggesting slight changes in the DBR properties over multiple sensing cycles.

**Figure 5:**
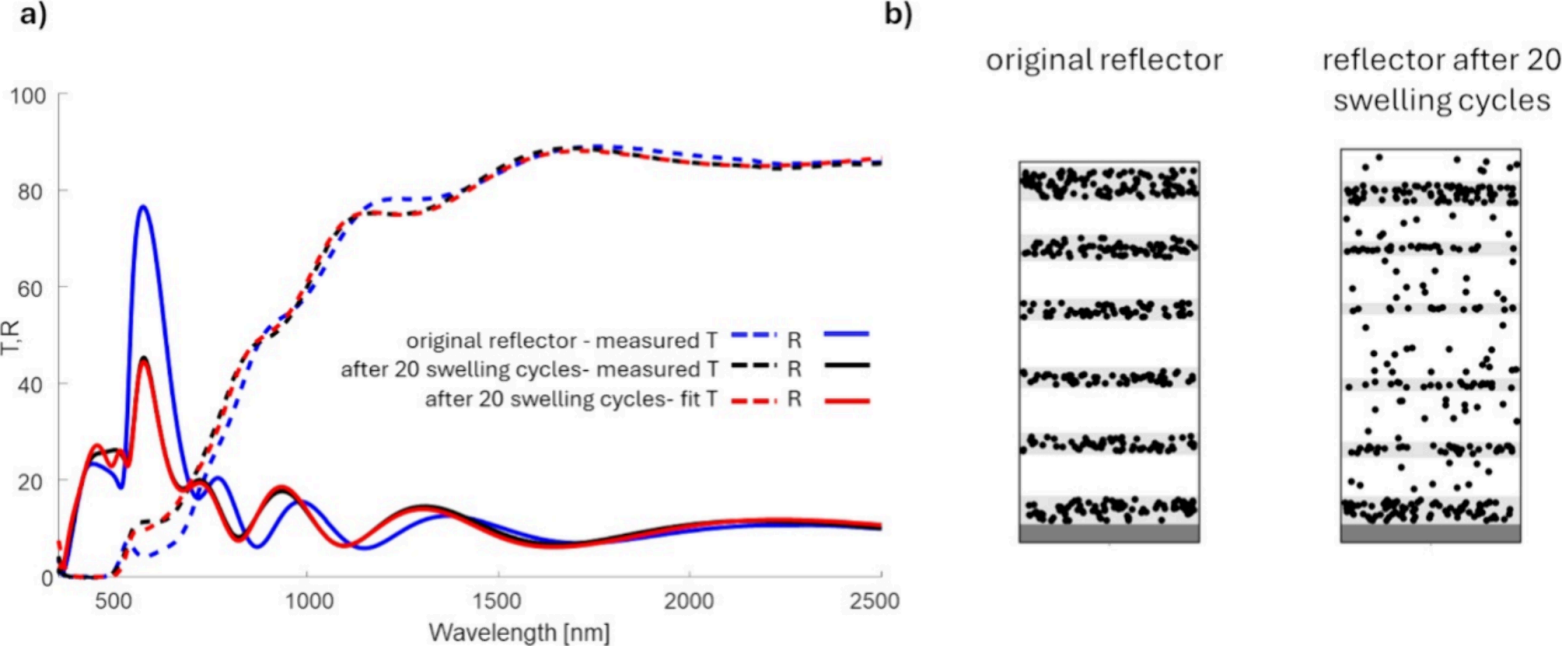
(a) Transmission (*T*) and reflection (*R*) of the DBR after 20 cycles of swelling and collapsing; black lines: measured *T* and *R*; red lines: *T* and *R* fitted with the GMG-EMA optical model; blue lines: *T*,*R* of the original reflector. (b) Schematic structure of the original reflector and reflector after 20 swelling cycles.

To assess the DBR’s long-term stability, we subjected it to 20 cycles of ethanol vapor exposure, followed by argon purging. The swelling and deswelling behaviors remain consistent across cycles; however, the reflectance intensity progressively degrades with each cycle. This degradation is illustrated in Figure 4a, where the maxima for each cycle, in both swollen and nonswollen states, are incrementally lower than the previous cycle. The greatest loss in performance occurred during the first swelling cycle, with a decrease of *R* intensity by 4%. Each subsequent cycle resulted in an additional performance loss between 1 and 2%. After 20 swelling cycles, the *T* and *R* spectra, shown as black lines in Figure 5a, reveal a significant decrease in reflectance from 77% in the initial state (blue line) to 47% after cycling (black line). The intensity drop indicates a decrease in the refractive index contrast. The minimal change in the peak position suggests that the optical thickness of the bilayers (*n*_cl_ × *d*_cl_ + *n*_pl_ × *d*_pl_) remains nearly the same, despite a slight blue shift in the entire spectrum. This outcome can result from a reduced distinction between the optical properties of the high- and low-refractive-index layers. Such changes are likely caused by the diffusion of nanoparticles (NPs) from the composite layers into the low-refractive-index polymer layers, which diminish the optical contrast between these layers. It is important to emphasize a key simplification in the employed model: in reality, the structure more closely resembles a rugate filter, as the interfaces between the high- and low-refractive-index layers are no longer sharply defined. Consequently, the described NP diffusion refers to changes in the NP density profile within the structure rather than a distinct migration. The fit of the optical model is shown as red lines in Figure 5a.

Figure 5b schematically illustrates the modeled structures before and after ethanol cycling. The high-filling factor layers show reduced thickness compared to the original reflector, yet their positions within the stack remain consistent, retaining approximately 16% volumetric filling factor of silver, which is the same as in the original reflector. For the low filling factor layers (originally just polymer films with an initial Ag filling factor of 0%), a new filling factor of around 2% was determined, indicating substantial structural changes from repeated swelling and recovery cycles. As the EMA models assume volumetric filling factors, the thickness of the composite layers, along with the filling factor, provides insights into the total effective silver content within the structure:
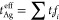
8

The *t*_Ag_^eff^ can be imagined as an equivalent silver thickness. The amount of silver in the original reflector was estimated as being equivalent to 55 nm, while after swelling cycles, the total amount of silver in both high and low filling factor composites is 53 nm. This good agreement between the amount of silver in the original reflector and the degraded one supports the validity of our model, indicating that the structural changes observed in the reflector are well-captured by this approach. We attempted to verify our model by taking cross-sectional SEM images before and after ethanol sensing and comparing them. Although a visual difference is observed, the evidence remains ambiguous due to difficulties in SEM measurements following the degradation of the reflector under the electron beam, the presence of charging, etc. (see the Supporting Information and Figure S3).

**Figure 6:**
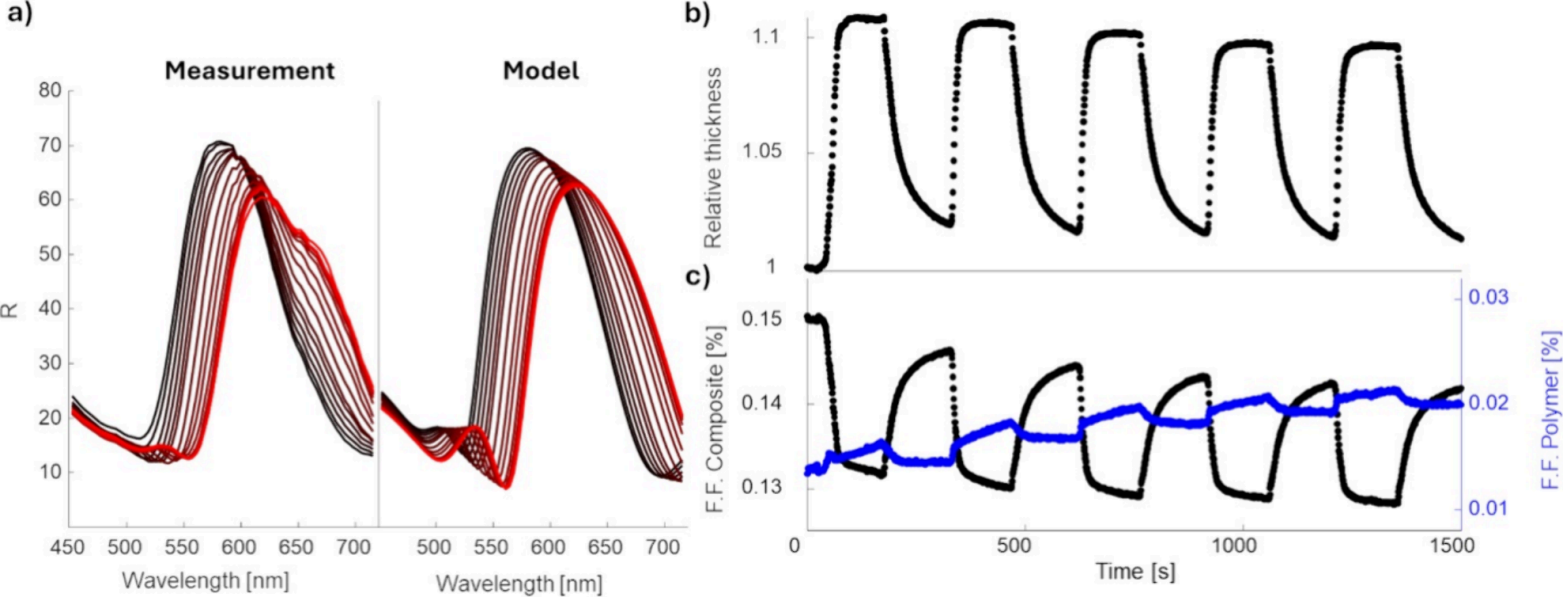
(a) Measured and fitted structure of the DBR during the first ethanol exposure swelling. (b) Relative thickness of the whole DBR structure, obtained via optical modeling, and (c) Filling factor of the composite layers and of the polymer layers as a function of cycling.

Considering that the sensing mechanism relies on the swelling of the structure and that degradation is caused by a blending of the composite structure, we attempted to create an optical model for the in situ reflection spectra phenomenologically described earlier and shown in Figure 4. However, compared to the spectra measured before and after exposure, the in situ data offer limited information—specifically, a narrower spectral range and the absence of transmission data. Additionally, the in situ reflection spectra differ slightly from those obtained using full-range UV–vis–NIR on a different spectrophotometer. Therefore, we opted to keep the model as simple and constrained as possible, aiming at capturing only the effective behavior rather than a perfect spectral match. The model is compared with the measured spectra during the first swelling cycle in Figure 6a. The rest of the swelling cycles are shown in Figure S4.

In the in situ model, we assume that the structure, specifically, the relative thickness of each individual layer and the nanoparticle properties within each layer, remains the same as in the original model describing the reflector before the swelling test. We further assume that all layers within the entire structure swell at a uniform rate *x*_1_. This assumption is based on the fact that the composite layers contain more than 80% of polymer. Therefore, we assume that they behave similarly to a pure polymeric layer. Although the assumption of equivalent swelling across all layers may not be entirely accurate, introducing additional parameters does not yield any significant improvements to the fit. Let the thickness of layer *i* at time *t* be expressed as
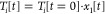
9

As the amount of silver within each layer remains constant, the filling factor decreases as the layers are swelling:
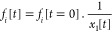
10

With this approach, a single cycle of swelling can be reasonably well-fitted; however, it does not account for any degradation mechanisms. To incorporate degradation, we assume that silver nanoparticles migrate from the composite layers toward the polymeric layers. This is the same simplification as that in the fitting of the final reflector.

According to previous works focused on the mechanism of NP diffusion within polymer media,^42^ there are multiple pathways to the enhanced NP migration resulting in a decrease of optical contrast and sensing performance degradation:

1. Partially dissolving polymer chains can increase particle diffusion by temporarily reducing the density and rigidity of the topological constraints, such as network cages and entanglement nets, around the particles. This allows more frequent fluctuations in the confinement structure.
2. The softened and more dynamic polymer environment lowers the free energy barrier for hopping diffusion, enabling particles to move between neighboring cages with less resistance and a shorter waiting time for favorable cage deformations.
3. In systems where chain dissolution reduces the network mesh size or weakens cross-links, the hopping step size remains small, but the effective diffusion coefficient increases due to the enhanced mobility of polymer strands, which improves the slippage mechanism around large particles.

By applying cyclic stretching of the host matrix by swelling of the structure, this can result in effective diffusion of the nanoparticles from a high-concentration region—nanocomposite layers—toward a low concentration region—polymeric layer—effectively blurring the difference between them.

By abstracting the continuous microstructural changes with rigid layers and interfaces, we model the changes depicted in Figure 6a between the original and final states of the reflector. We assume that the amount of silver within the whole structure is constant.
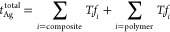
11

Therefore, the model consists of a second parameter *x*_2_, which represents the effective thickness of silver removed from the composite and incorporated into polymer layers, while
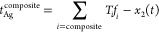
12
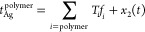
13

The filling factor of both the composite and polymeric layers is updated at each step based on the total amount of silver, as calculated in eqs 12 and 13, and the degree of swelling, as determined by eqs 9 and 10. The evolution of filling factors is shown in Figure 6b,c.

The advantage of this approach lies in the straightforward interpretability of the fitted parameters. Parameter *x*_1_ represents the swelling ratio of the entire structure, while *x*_2_ describes the migration of nanoparticles from the dense composite toward the polymeric layers. This migration leads to a decrease in the contrast between the layers of low and high refractive index.

## 4 Conclusion

This work presents a proof of concept for DBR sensors incorporating plasmonic silver nanoparticles embedded in a PLA-like plasma polymer matrix. While the synthesized DBR demonstrates promising sensing capabilities, the ethanol vapor concentrations tested are relatively high. Although the sensor shows a significant and rapid response, indicating the potential for detecting lower VOC concentrations, practical applications would require higher sensitivity. Additionally, the sensor undergoes rapid degradation. Nonetheless, we believe that there is room for improvement. For instance, tuning the plasma-polymer-like properties of the matrix via the PAVTD method could improve the probable trade-off between the sensitivity of the sensor and its stability. Lowering the VOC concentration could also help mitigate the degradation. In all three cases of the optical modeling—before swelling, in situ, and after swelling—the reflector is fitted using a highly simplified model. Nevertheless, this optical model allowed us to establish the core mechanisms of sensing—swelling of the structure by 10%—as well as a core mechanism of degradation—blurring of the structure, where nanoparticles do not return to their original positions. We have also found that the reflector is sensitive to polar organic and nonorganic vapors while nonsensitive to nonpolar volatile organic compounds.

In addition, important key insights from this work can be derived. First, plasmonic nanoparticles, particularly silver, offer an effective approach for significantly increasing the refractive index of materials without necessarily compromising properties such as permeability and swellability. In this setup, we achieved a refractive index close to 2.0 using a silver volumetric filling factor of approximately 15%. It is important to recognize that this high refractive index is accompanied by absorption in the blue part of the visible spectrum and by a highly nonlinear spectral dependence of the refractive index. However, in the case of a DBR, this is not necessarily a counterproductive feature if we do not aim for close to 100% reflectivity.

Second, the GMG-EMA optical modeling approach for plasmonic nanocomposites shows excellent agreement with the experimental results. While plasmonic composites have been widely studied over the past two decades using various effective medium approximation models, such as GMG, MG, and Bruggeman, this approach has not yet been extensively applied to the design and analysis of complex optical filters. We demonstrate that GMG-EMA can be effectively used for detailed investigations of plasmonic composites and complex structures. Such an approach can bring insight into active plasmonic nanocomposite-based optical devices, such as the present reflector or stretch sensors,^43^ as well as passive devices, such as plasmonic absorbers,^44^ antireflective coatings,^45^ and transparent electrodes.^46^

## Supporting Information Available

Schematic of the VOC sensing setup, fit of the transmission and reflection spectra of the reflector with the MG-EMA optical model, SEM of the reflector before and after sensing, and comparison of measured spectra with the model for all five sensing cycles (PDF)
