# Supplementary material for: Sensing of Organic Vapors with Plasmonic Distributed Bragg Reflectors
Source: ACS Appl Mater Interfaces. 2025 Apr 24;17(18):27126–35. doi: 10.1021/acsami.5c02058 (PMC12067376; doi:10.1021/acsami.5c02058)

Supporting Information

Sensing of Organic Vapors with Plasmonic Distributed Bragg Reflectors

[1,2] Zdeněk Krtouš*, [3] Oleksandr Polonskyi, [2] Pavel Pleskunov, [4] Miroslav Cieslar, [2] Bill Baloukas, [2] Ludvik Martinu, [1,5]Jaroslav Kousal

[1] Department of Macromolecular Physics, Faculty of Mathematics and Physics, Charles University, V Holešovičkách 2, 180 00, Prague, Czech Republic

[2] Department of Engineering Physics, Polytechnique Montréal, Montreal, QC H3T 1J4, Canada

[3] Department of Chemical Engineering, University of California, Santa Barbara, CA 93106-5080, USA

[4] Department of Physics of Materials, Faculty of Mathematics and Physics, Charles University, Ke Karlovu 5, Prague, 121 16, Czech Republic

[5] Department of Aerospace Engineering, Faculty of Mechanical Engineering, Czech Technical University in Prague, Karlovo náměstí 13, 121 35, Prague, Czech Republic

* Corresponding author

E-mail address: krtousz@gmail.com (Z. Krtouš)

Figure S1. VOC Sensing Setup

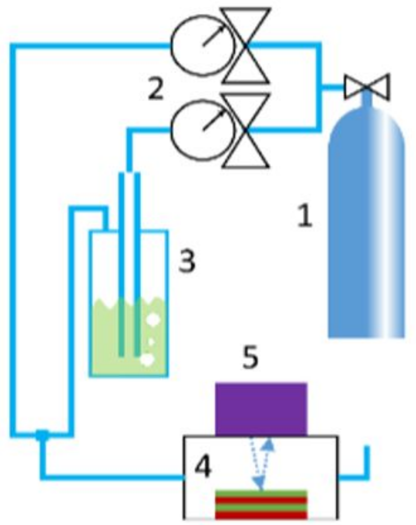

1 – gas tank; 2 –flow controllers; 3 – bubbler with test chemical; 4 – flow cell with the sample; 5 – UV/VIS spectrophotometer

Figure S2. Maxwell Garnett optical model of DBR

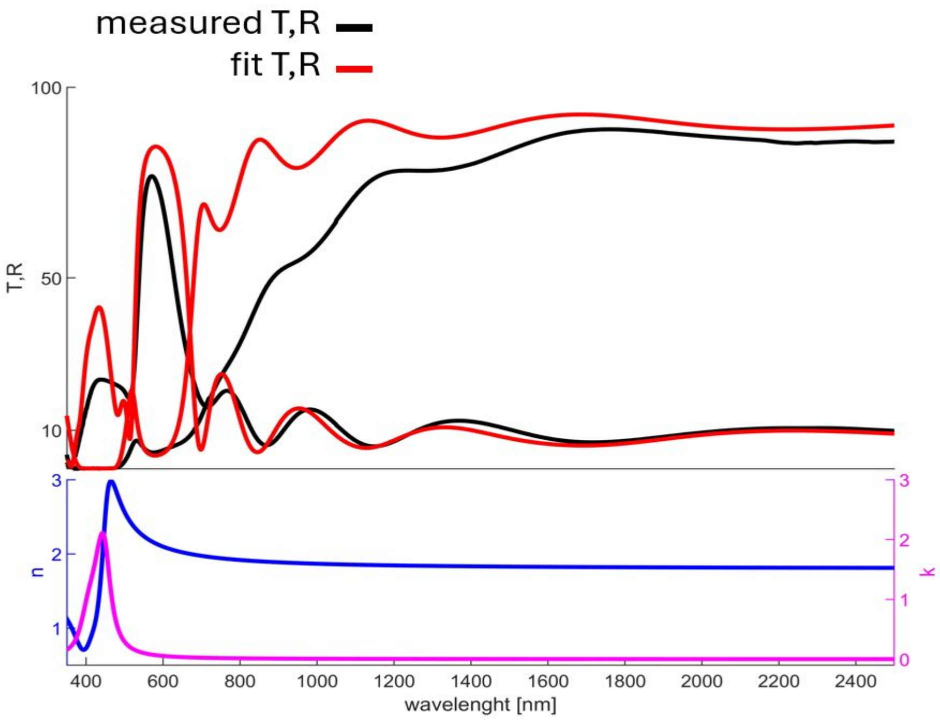

Figure S3. SEM before and after sensing

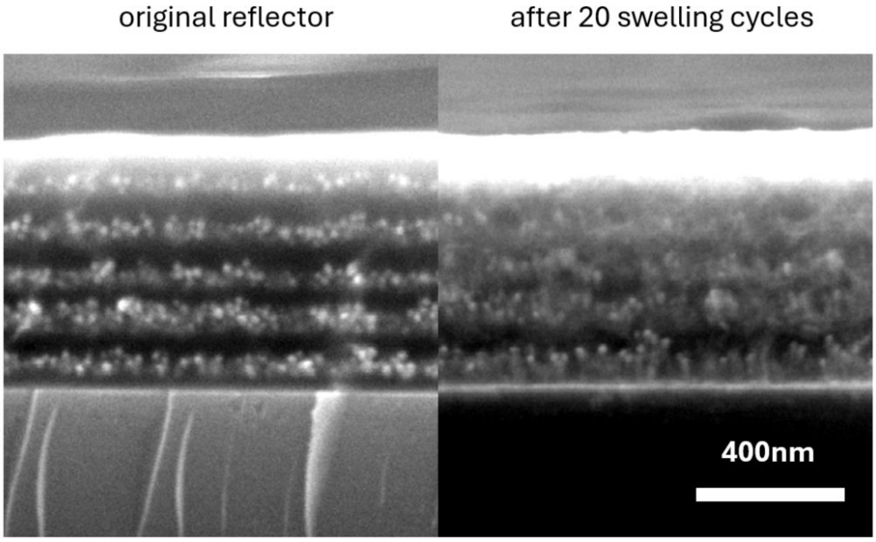

Figure S4. Comparison of Measurement and Optical Model of Swelling

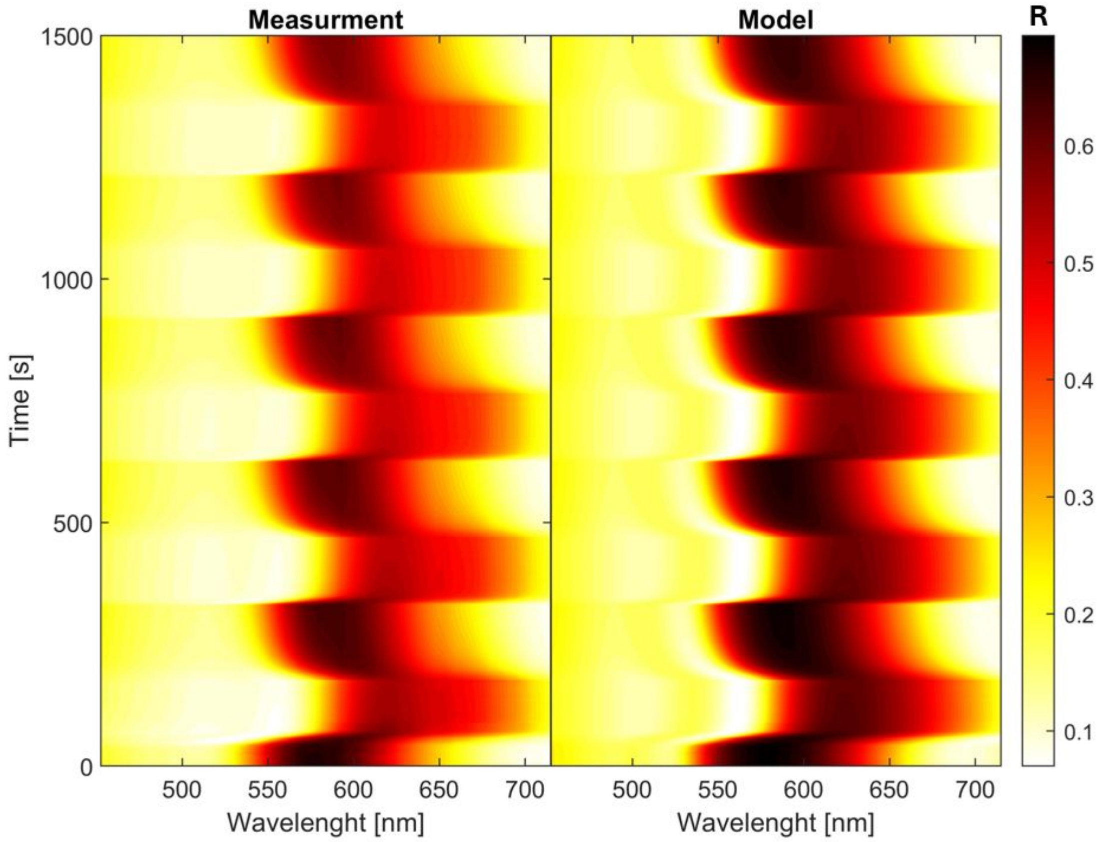

Supplement: Supplementary file 1 — am5c02058_si_001.pdf [file am5c02058_si_001.pdf]
